# Lighter Ingestion as an Uncommon Cause of Severe Vomiting in a Schizophrenia Patient

**DOI:** 10.1155/2016/6301302

**Published:** 2016-07-21

**Authors:** Yahya Atayan, Yasir Furkan Cagin, Mehmet Ali Erdogan, Yılmaz Bilgic, Remzi Bestas, Murat Harputluoglu, Yüksel Seckin

**Affiliations:** ^1^Department of Gastroenterology, Gümüşhane State Hospital, 29100 Gümüşhane, Turkey; ^2^Department of Gastroenterology, Medical Faculty, Inonu University, Malatya, Turkey; ^3^Department of Gastroenterology, Diyarbakir Education and Researches Hospital, Diyarbakir, Turkey

## Abstract

*Background*. Foreign bodies in the gastrointestinal tract are important morbid and mortal clinical conditions. Particularly, emergency treatment is required for cutting and drilling bodies. The majority of ingested foreign bodies (80–90%) leave gastrointestinal tract without creating problems. In 10–20% of cases, intervention is absolutely required. Less than 1% of cases need surgery. In this paper, we present a schizophrenia patient who swallowed multiple lighters.* Case*. A 21-year-old male schizophrenic patient who uses psychotic drugs presented to the emergency department with the complaints of abdominal pain, severe vomiting, and inability to swallow for a week. His physical examination revealed epigastric tenderness. A plain radiograph of the abdomen revealed multiple tiny metallic densities. Gastroscopy was performed. The lighters were not allowing the passage, and some of them had penetrated the gastric mucosa, and bezoars were observed. One lighter was extracted with the help of the polypectomy snare. Other lighters as a bezoar were removed by surgery.* Conclusion*. Excessive vomiting of swallowed foreign bodies in the etiology of psychotic patients should be kept in mind. Endoscopic therapy can be performed in the early stages in these patients, but in the late stage surgery is inevitable.

## 1. Introduction

Foreign bodies are generally ingested accidentally when eating other foods [1]. Foreign body ingestion is a mortal and morbid clinical condition. It is frequent in children, the elderly, and prisoners, and psychotic cases follow this group. It most frequently occurs in schizophrenic patients in psychotic cases [2]. Foreign bodies mostly tend to get stuck in the esophagus and the stomach. The esophagus has three areas of narrowing: the upper esophageal sphincter, the level of the aortic arch, and the diaphragmatic sphincter. More than 80% of foreign bodies often pass through the gastrointestinal tract without any intervention. Furthermore, foreign objects larger than 6 cm cannot pass the physiological narrowing of the pylorus and remain in the stomach. We hereby present a schizophrenic patient who had presented to the emergency department with the complaint of sudden onset of severe vomiting after having ingested lighters and a plum.

## 2. Case Presentation

**Figure 1:**
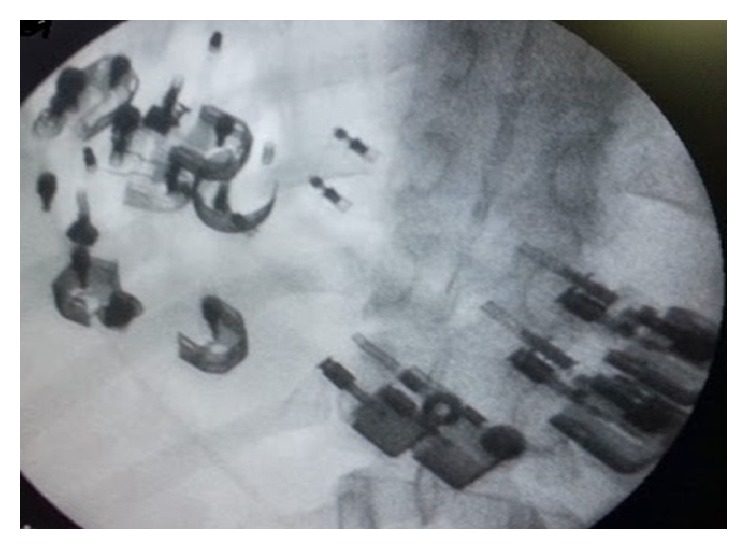
The plain abdominal radiograph showing lighters.

**Figure 2:**
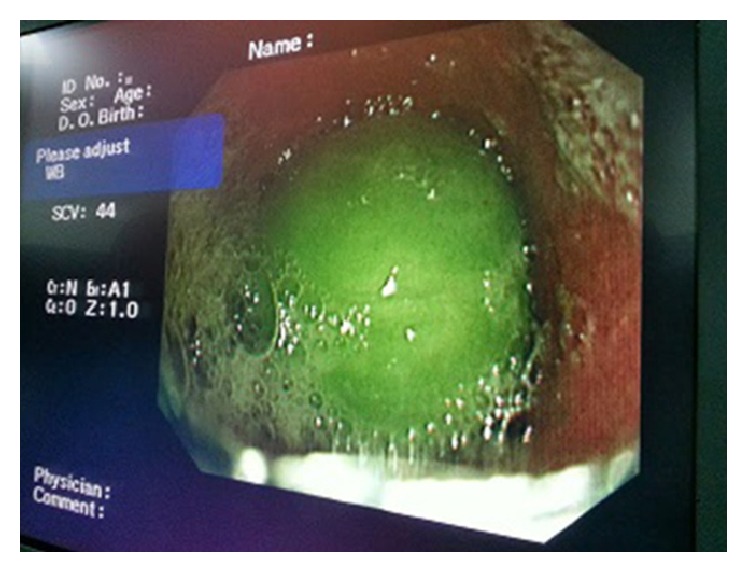
Gastroscopic view showing a plum causing complete obstruction.

**Figure 3:**
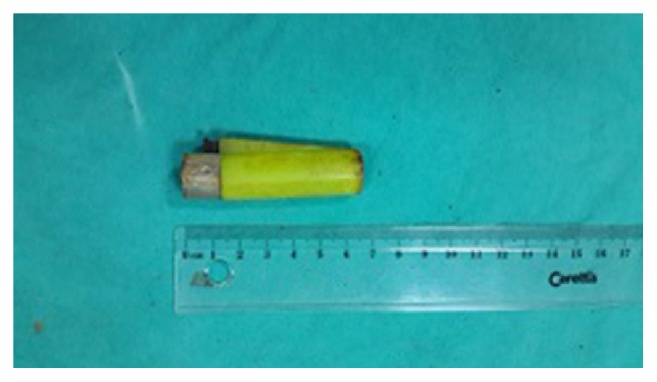
The lighter extracted.

A 21-year-old male patient who had visual hallucinations and behavioral and cognitive disorders presented to the psychiatry clinic and was diagnosed with moderate mental retardation (patients were mismatched to The* Wechsler* Adult Intelligence Scale, IQ in the 39–49 range) and schizophrenia. Risperidone was prescribed by the psychiatrist at 2 mg/day. He presented to the emergency department with the complaints of abdominal pain, severe vomiting, and inability to swallow for a week. Physical examination revealed epigastric tenderness without defense or rebound. The laboratory values were as follows: white blood cells 5 000/mm^3^, hemoglobin 12 g/dL, platelets 155 000/mm^3^, glucose 78 mg/dL, BUN 55 mg/dL, creatinin 1.1 mg/dL, sodium 138 mEq/L, and potassium 4.0 mEq/L. A plain radiograph of the abdomen revealed multiple tiny metallic densities (Figure 1). The patient underwent the consultation of our gastroenterology service. He was hospitalized and a gastroscopy was performed. A plum that had caused complete obstruction was detected at the level of the diaphragmatic hiatus, and it was seen to have been pushed into the stomach (Figure 2). An ulcer in the esophagus and lighters of different sizes that filled the lumen of the gastric corpus and antrum were observed. The lighters were not allowing passage, and some of them had penetrated the gastric mucosa, and bezoars were observed. A lighter was extracted with the help of the polypectomy snare (Figure 3). Extraction of the other foreign bodies was not possible because of bezoars and penetration into the gastric mucosa. Twelve lighters were extracted by general surgery. The patient was operated on and the 12 lighters were removed. Postoperatively, the patient underwent the consultation of the psychiatry clinic, and the patient's swallowing of the lighters was associated with impulsive behavior due to mental retardation. He was then admitted to the psychiatric clinic for therapy.

## 3. Discussion

Foreign bodies are generally ingested accidentally in adults, usually in psychotic patients and prisoners. Healthy adults and older children can typically identify foreign body ingestion and may point to a specific area of discomfort, though the area usually does not correspond to the impacted side [3]. However, children, mentally impaired adults, and patients with psychiatric illness may not give a history of foreign body ingestion. In these cases, we should consider foreign body ingestion due to symptoms. Symptoms may appear anywhere from minutes to years after ingestion. Foreign bodies can be organic or inorganic according to their structure and blunt or sharp according to trauma-forming properties. Psychiatric patients can ingest foreign bodies of different sizes and shapes. The incidence of ingesting lighters is unknown. The case is extremely rare in the literature. Trgo et al. [4] have reported a case in which a lighter was extracted by endoscopy. In our case, the patient had ingested twelve lighters; one of them was extracted by endoscopy, and the others were extracted by surgery due to the complication risks. Foreign bodies most frequently tend to adhere to the esophagus and the stomach. The esophagus has three areas of narrowing: the upper esophageal sphincter, the level of the aortic arch, and the diaphragmatic sphincter. Complications of foreign body ingestion or food impaction include ulcer formation, lacerations, perforation, intestinal obstruction, aortoesophageal fistula formation, tracheoesophageal fistula formation, and bacteremia [5]. More than 80 percent of foreign bodies often pass through the gastrointestinal tract without any intervention. However, endoscopic intervention is definitely required in 10–20% and surgical intervention is required in less than 1% of the patients with intentional foreign body ingestion. Toxic materials such as hydrocarbons, benzene, butane, hexamine, and propane cause gastric ulcer and complications with corrosive effects. The symptoms that may appear after ingestion of foreign bodies include difficulty in swallowing, even that of saliva at the early period [6]. Dysphagia occurs in 60–95% of the cases and tenderness in the neck region occurs in 50–65% of the cases [7]. Radiographic examination should always be performed in foreign body ingestion. Some foreign bodies cannot be seen on direct radiography. In these cases, bilateral radiography should be performed. If nothing is seen on radiographs and there is a suspicion, endoscopic procedures can be performed for the diagnosis and treatment [8]. Flexible endoscopic examination should be performed for the diagnosis and treatment. Extraction of larger foreign bodies depends on the technical abilities of the endoscopist and the clinical condition of the patient. If endoscopic extraction is unsuccessful or there is evidence of obstruction and perforation, surgical gastrostomy should be performed. In our case, surgical gastrostomy was inevitable, because there was an ulcerated obstruction and bezoar made of foreign bodies. Flexible and rigid endoscopies are both successful in more than 90 percent of the cases, but rigid endoscopy has a higher perforation rate [8, 9]. However, the overall incidence of esophageal perforation with these techniques is low. In a series of 192 adults and children requiring foreign body extraction with rigid or flexible esophagoscopy, no serious complication (mediastinitis, hemorrhage, perforation, or death) was noted [9]. In a second study, perforation occurred in 2 of 63 patients (3 percent) who had undergone rigid endoscopy, and no perforation occurred in 76 patients who had undergone flexible endoscopy [8]. Devices that are commonly used for foreign body removal included rat-tooth and alligator forceps, polypectomy snares, baskets, and nets. The rat-tooth forceps and the snare are the most frequently used devices. If endoscopic extraction is unsuccessful or there is evidence of obstruction or perforation, surgical gastrostomy should be performed. In our case, a lighter was extracted with the help of the endoscopic polypectomy snare (Figure 3). Extraction of the other foreign bodies was not possible due to presence of bezoars and penetration into the gastric mucosa. Twelve lighters were extracted through surgery.
